# Population-level seropositivity trend for SARS-Cov-2 in Rio Grande do Sul, Brazil

**DOI:** 10.11606/s1518-8787.2021055004075

**Published:** 2021-11-08

**Authors:** Aluísio J D Barros, Cesar G Victora, Ana M B Menezes, Bernardo L Horta, Fernando C Barros, Fernando P Hartwig, Gabriel D Victora, Luis Paulo Vidaletti, Mariângela F Silveira, Marilia A Mesenburg, Nadège Jacques, Cláudio J Struchiner, Flávia Roberta Brust, Marinel M Dall’Agnol, Ana Paula Longaray Delamare, Carlos Henrique R François, Maria Letícia R Ikeda, Débora C P Pellegrini, Cézane Priscila Reuter, Shana G da Silva, Odir A Dellagostin, Pedro C Hallal

**Affiliations:** I Universidade Federal de Pelotas Faculdade de Medicina Programa de Pós-Graduação em Epidemiologia PelotasRS Brasil Universidade Federal de Pelotas. Faculdade de Medicina. Programa de Pós-Graduação em Epidemiologia. Pelotas, RS, Brasil; II The Rockefeller University Laboratory for Lymphocyte Dynamics New YorkNY USA The Rockefeller University. Laboratory for Lymphocyte Dynamics. New York, NY, USA; III Fundação Getúlio Vargas Escola de Matemática Aplicada Rio de JaneiroRJ Brasil Fundação Getúlio Vargas. Escola de Matemática Aplicada. Rio de Janeiro, RJ, Brasil; IV Universidade Federal de Ciências da Saúde de Porto Alegre Departamento de Saúde Coletiva Porto AlegreRS Brasil Universidade Federal de Ciências da Saúde de Porto Alegre. Departamento de Saúde Coletiva. Porto Alegre, RS Brasil; V Universidade Federal de Santa Maria Centro de Ciências da Saúde Departamento de Saúde Coletiva Santa MariaRS Brasil Universidade Federal de Santa Maria. Centro de Ciências da Saúde. Departamento de Saúde Coletiva. Santa Maria, RS, Brasil; VI Universidade de Caxias do Sul Instituto de Biotecnologia Programa de Pós-Graduação em Biotecnologia Caxias do SulRS Brasil Universidade de Caxias do Sul. Instituto de Biotecnologia. Programa de Pós-Graduação em Biotecnologia. Caxias do Sul, RS, Brasil; VII Universidade Regional do Noroeste do Estado do Rio Grande do Sul Faculdade de Medicina IjuíRS Brasil Universidade Regional do Noroeste do Estado do Rio Grande do Sul. Faculdade de Medicina. Ijuí, RS, Brasil; VIII Universidade do Vale do Rio dos Sinos Programa de Pós-Graduação em Saúde Coletiva São LeopoldoRS Brasil Universidade do Vale do Rio dos Sinos. Curso de Enfermagem. Programa de Pós-Graduação em Saúde Coletiva. São Leopoldo, RS, Brasil; IX Universidade Federal do Pampa Programa de Pós-Graduação em Ciência Animal UruguaianaRS Brasil Universidade Federal do Pampa - Campus Uruguaiana. Programa de Pós-Graduação em Ciência Animal. Uruguaiana, RS, Brasil; X Universidade de Santa Cruz do Sul Departamento de Ciências da Saúde Programa de Pós-Graduação em Promoção da Saúde Santa Cruz do SulRS Brasil Universidade de Santa Cruz do Sul. Departamento de Ciências da Saúde. Programa de Pós-Graduação em Promoção da Saúde. Santa Cruz do Sul, RS, Brasil; XI Universidade Federal da Fronteira Sul Programa de Pós-Graduação em Ciências Biomédicas Passo FundoRS Brasil Universidade Federal da Fronteira Sul. Curso de Medicina. Programa de Pós-Graduação em Ciências Biomédicas. Passo Fundo, RS, Brasil

**Keywords:** COVID-19, epidemiology, Seroepidemiologic Studies, Immunity, Herd, Socioeconomic Factors, Health Surveys

## Abstract

**OBJECTIVE:**

To describe the evolution of seropositivity in the State of Rio Grande do Sul, Brazil, through 10 consecutive surveys conducted between April 2020 and April 2021.

**METHODS:**

Nine cities covering all regions of the State were studied, 500 households in each city. One resident in each household was randomly selected for testing. In survey rounds 1–8 we used the rapid WONDFO SARS-CoV-2 Antibody Test (Wondfo Biotech Co., Guangzhou, China). In rounds 9–10, we used a direct ELISA test that identifies IgG to the viral S protein (S-UFRJ). In terms of social distancing, individuals were asked three questions, from which we generated an exposure score using principal components analysis.

**RESULTS:**

Antibody prevalence in early April 2020 was 0.07%, increasing to 10.0% in February 2021, and to 18.2% in April 2021. In round 10, self-reported whites showed the lowest seroprevalence (17.3%), while indigenous individuals presented the highest (44.4%). Seropositivity increased by 40% when comparing the most with the least exposed.

**CONCLUSIONS:**

The proportion of the population already infected by SARS-Cov-2 in the state is still far from any perspective of herd immunity and the infection affects population groups in very different levels.

## INTRODUCTION

In Brazil, the first case of COVID-19 was reported on 27 February 2020 in the city of São Paulo, and by mid-May 2021 the country had had over 15 million confirmed cases and 420 thousand deaths, the second highest cumulative mortality in the world. In March 2021, Brazil had a spike in cases and deaths that brought health services to the brink of collapse, with lack of oxygen and respirators in the North region. The daily number of new cases reached nearly 80 thousand, some 360 new cases per million people^1^. However, different from other countries that had huge spikes in cases, like Italy, the USA, and the UK with up to 880 new cases per million people, Brazil did not have a sharp decrease after the spike. Compared to these countries, only the US had had more cases per million people than Brazil.

Rio Grande do Sul, the southernmost state in Brazil, and the Southern region, in general, had a slow start in the pandemic compared to other regions of the country, but the number of new cases rose sharply in December 2020, and spiked in March 2021, when the number of new cases was close to 800 per million people. This increase coincided with the dissemination of the P.1 variant. Since then, despite the decline in the number of new cases, it remains high, at around 350 new cases per million, close to the country’s average. We summarized these indicators from the Brazilian Ministry of Health (https://covid.saude.gov.br/) and the State Secretary of Health (https://ti.saude.rs.gov.br/covid19/) on 11 May 2021.

However, the number of confirmed cases is much lower than the total number of infections in the population, making studies like the EPICOVID19-RS that monitors the percentage of the population that has already been infected with SARS-Cov-2 essential to understand the pandemic dynamics. Only a few nationwide seroprevalence studies have been reported, mostly in European countries during the first months of the pandemic. In April 2020, 0.8% (0.6–1.0) tested positive for SARS-CoV-2 antibodies in Iceland^2^, and 0.33% (0.12–0.76) in Austria^3^. In Spain, 5.2% (4.9–5.5) of the populations were seropositive in June 2020^4^.

In Brazil, we reported seroprevalence levels of 1.9% (1.7–2.2) for May 2020 and 3.1% (2.8–3.4) for June 2020, from a national seroprevalence study^5^. A study in the state of Maranhão, in northern Brazil, collecting data in selected areas, found a much higher seroprevalence of 38.1% (34.8%–41.1%)^6^. Another study based on samples from blood donors in the city of Manaus in the state of Amazonas estimated that between 44% and 66% had already been infected with SARS-Cov-2 by July 2020^7^. This raised the possibility that the city was close to reaching herd immunity, which was soon dismissed by another spike in cases, as the same research team discussed. Overestimation of the population seropositivity that was based on blood donors, population mobility and the appearance of coronavirus variants in the population may explain the second wave^8^. Since then, hopes of herd immunity ending the epidemic – an idea repeatedly suggested by Brazil’s president^9^ – seem to have waned.

Intending on monitoring the population level of seropositivity we started the EPICOVID19-RS study in April 2020 and by September we had conducted eight rounds of the study. Seroprevalence was estimated by using the Wondfo rapid test, increasing from 0.03% in round 1 to 1.89% in round 8^10^. With time, we noticed that the test’s sensitivity could not be as high as we initially believed and that it decreased over time^11^. Therefore, we sought for a better test and for ways to correct the estimates already available.

After a 5-month pause since the eighth round of the EPICOVID19-RS study, we carried out a ninth round then using a new ELISA test^12^, alongside the Wondfo rapid test, and a tenth round soon after. The these rounds aimed to estimate the population prevalence of seropositivity for SARS-Cov-2 for the whole sample and for population subgroups defined by age, sex, ethnicity, and wealth. We also studied social distancing measures and vaccination status.

## METHODS

The EPICOVID19-RS study started just 18 days after the first COVID-19 death in the state, to monitor population SARS-Cov-2 infection level. So far, 10 rounds of the population-based survey were completed, the first in April 2020 and the last in April 2021. A similar sampling methodology was used in all rounds. We used a multistage sampling approach based on nine sentinel cities^13^.

In the first nine rounds of the study, we used the rapid point-of-care lateral-flow WONDFO SARS-CoV-2 Antibody Test (Wondfo Biotech Co., Guangzhou, China), which can detect both IgM and IgG antibodies. The Wondfo test manufacturer rates the test sensitivity and specificity at 86.4% and 99.6%, respectively (URL: https://www.bilcare.com/SARS-CoV-2%20Antibody%20Test%20(Lateral%20Flow%20Method).pdf, accessed 11 May 2021). We conducted two separate validation studies on this test. In the first, we estimated a sensitivity of 84.8% with recently diagnosed patients^13^. In the second, we enrolled 133 patients who had had positive RT-PCR results from a period of a few days up to six months before. Here we found sensitivities varying from around 80% (among subjects diagnosed in the previous two months) to 42% for the earliest diagnosed patients^11^. The test sensitivity observed in the previous validation was confirmed for recent infections, but sensitivity decreased with time. Therefore, we developed a method to adjust the seroprevalences obtained with the Wondfo test starting by using the number of deaths as an indicator of the temporal distribution of the epidemic (data available from the state of Rio Grande do Sul COVID-19 information committee). The second step was to estimate a function describing the sensitivity decay for the Wondfo test. This calibration procedure ensures the sensitivity function is more coherent with field estimates of sensitivity, which may differ from estimates in the validation study. To calculate the adjusted Wondfo prevalence estimates in rounds 1–8, sensitivity was calculated as the average of the sensitivity function weighted by the daily number of deaths up to the date of each survey. Details of the correction procedure are described elsewhere^14^.

In round nine, we also used an in-house direct ELISA test that identifies the presence of IgG to the viral spike (S) protein from dried blood spot samples (S-UFRJ) in parallel with the Wondfo test. The developers estimated the test specificity to be 98.6%, and sensitivity 95.0% (binomial 95%CI 92.3–97.0)^15^. Our validation of this test, only with participants that were positive in a RT-PCR test diagnosed up to six months before the study, revealed a sensitivity of 92.5% (95%CI 86.6–96.3)^11^. Given the small impact of correcting for these values of sensitivity and specificity at this prevalence level, we opted for presenting the unadjusted ELISA estimates. The ELISA test was processed in our own laboratory according to the developer specification. In round ten, only the ELISA test was used. In both rounds, the participants were informed of their own results as soon as the ELISA analyses were completed.

A short questionnaire including information on sex, age, schooling, self-reported skin color and compliance with social isolation measures was applied after testing. Schooling was recorded as the highest year completed successfully. The IBGE categories were used to classify subjects by their skin color (or ethnicity). Individuals were asked to self-classify into white, brown (“*pardo*” in Portuguese), black, yellow or Asian, or indigenous. Ownership of a series of assets was recorded to assess household wealth from round four^16,17^. The assets were: automobile for personal use, desktop or notebook computer, color TV, air conditioning, cable internet, cable TV, number of bathrooms and number of bedrooms in the house. Using these assets, we performed principal components analysis to extract the first component score and used it to classify households in terms of wealth, dividing them into five equally sized groups, the wealth quintiles (the first including the 20% poorest participants, up to the fifth which includes the 20% richest)^16,18^. The combined sample for the nine cities was used for deriving the asset score in each round.

In terms of social distancing, individuals were asked three questions: i) “To what extent are you managing to follow the social distancing guidance from the health authorities, i.e., staying at home and avoiding contact with others?”; ii) “What have your routine activities been?” with alternatives about the frequency of going out; and iii) “Thinking about the household routine, who has been in the house?” with alternatives about the presence of relatives and friends and its frequency.

Given that the three questions about individual and household routine inform on the exposure level of each participant, we used principal components analysis with the three variables to extract the scores for the first component that was used to indicate the individual exposure level. Score cut-offs were calculated to create 10 equally sized groups of participants, with the first group, D1, including the 10% least exposed, up to D10 with the 10% most exposed participants.

In rounds nine and ten we also asked about vaccination against SARS-CoV-2 – the participants intent on being vaccinated or whether they had already been vaccinated. The date of vaccination and the vaccine brand was also recorded.

All analyses took the sample design into account. Pooled seroprevalence estimates for cities and populations groups were not weighted by the size of each city and represent the average across the nine cities. For the last two rounds, seropositivity was based on the ELISA test, with the prevalence estimated directly from the observed results. For rounds one to eight, we used a correction strategy to adjust for the lower sensitivity of the Wondfo test for infections that occurred more than three months before.

For the last two study rounds, in which the ELISA test was available, we assessed the association between seroprevalence and the exposure score using a logistic regression model with the continuous exposure score, thus imposing a logit-linear relationship between the score and the outcome. Only unvaccinated individuals were used for fitting this model, whose goodness of fit was assessed by using the Hosmer-Lemeshow test. All the analysis were carried out with Stata (StataCorp. 2019. Stata Statistical Software: Release 16. College Station, TX: StataCorp LLC.) and with R (R Core Team (2020); R: A language and environment for statistical computing; R Foundation for Statistical Computing, Vienna, Austria).

All interviewers were tested for COVID-19 and only those with negative results and absence of any symptom worked in the field. In the last two rounds, interviewers who had been vaccinated or had COVID-19 more than a month before and had a negative RT-PCR test in the previous 30 days were also allowed to work. They all used individual protection equipment (masks, face shields, gloves, and aprons) that was discarded after visiting each household. Ethical approval was obtained from the Brazilian’s National Ethics Committee (30415520.2.0000.5313), and we obtained written informed consent from all participants. A separate informed consent form was used to obtain permission of parents or legally authorized representatives for minors. If the respondent was a child under age 12 or an older adult unable to answer the questionnaire, it was applied to the respondent’s legal guardian. Positive cases were reported to the statewide SARS-CoV-2 surveillance system.

## RESULTS

Along the 12 months and ten rounds of the study we observed fluctuations in the sociodemographic characteristic of the samples. Despite the statistical significance of some differences – given the large sample size, 44,611 in total – these fluctuations were not marked (Table 1). Females accounted for approximately 60% of the sample in all rounds. Children and adolescents were underrepresented, largely due to refusals likely caused by the finger prick. Apart from this, the percentages within each age group were stable. Most of the sample had incomplete or complete higher education. The ethnic composition of the sample was also stable along the rounds, with most participants self-classifying as white, followed by brown (“*pardo*”), and black. Individuals in the yellow or indigenous groups were few.

**Table 1:** Description of the sample in the ten survey rounds, April 2020 to April 2021, with rounds 1–8 presented together. Source: EPICOVID19-RS study, Brazil, 2020–21.

Variables	Survey round					

	1–8		9		10	
		
	n^a^	%	n^a^	%	n^a^	%
Sex (p = 0.0054)						
Male	14,327	40.2	1,731	38.5	1,725	38.3
Female	21,284	59.8	2,770	61.5	2,774	61.7
Age (years) (p < 0.0001)						
0–9	687	2.6	98	2.2	88	2.0
10–19	1,449	5.5	225	5.0	200	4.5
20–39	7,379	27.8	1,111	24.7	1,299	28.9
40–59	8,796	33.1	1,503	33.4	1,563	34.8
60–79	7,218	27.2	1,334	29.7	1,182	26.3
80+	1,055	4.0	228	5.1	159	3.5
Schooling (p < 0.0001)						
Primary (0–4 years)	1,758	4.9	232	5.6	187	4.6
Primary (5–9 years)	3,586	10.1	493	11.9	389	9.5
Secondary	4,530	12.7	586	14.1	535	13.1
Higher (incomplete)	9,804	27.6	1,223	29.4	1,290	31.6
Higher (complete)	15,907	44.7	1,626	39.1	1,688	41.3
Skin color (p = 0.0073)						
White	26,578	76.1	3,291	74.4	3,269	74.0
Brown (“*pardo*”)	5,455	15.6	708	16.0	716	16.2
Black	2,453	7.0	371	8.4	384	8.7
Yellow or Asian	262	0.8	29	0.7	28	0.6
Indigenous	173	0.5	26	0.6	18	0.4

^a^ Unweighted sample size for each subgroup.

**Figure 1:**
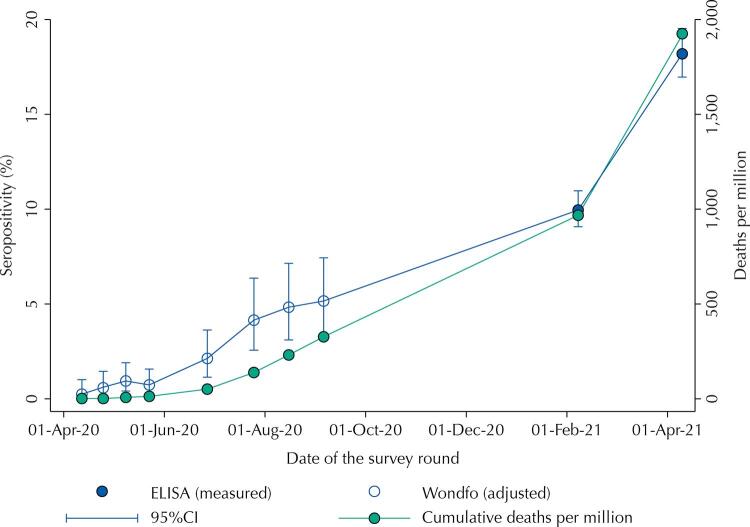
Seropositivity for the ten rounds of the EPICOVID19-RS study. Source: EPICOVID19-RS study, Brazil, 2020–21.

Table 2 shows the estimates of seropositivity (crude and adjusted) observed in the ten study rounds, along with the date, type of test and sample size. Antibody prevalence in early April 2020 was below 0.3% (0.26, 95%CI 0.03–1.00), increasing over time to 9.97% (95%CI 9.06–10.95) in February 2021, and to 18.16% (95%CI 16.90–19.49) in April 2021. Table 2 also shows the cumulative number of deaths per million people at each point. Figure 1 shows a graphical representation of the seropositivity time trend.

**Table 2:** Seropositivity in ten rounds of the EPICOVID19-RS study, along with dates, serologic test used, type of seropositivity estimation and sample size. Source: EPICOVID19-RS study, Brazil, 2020–21.

Study round	Median date	Sample size	ELISA	Wondfo rapid test		Cumulative deaths
		
			Crude	Crude	Adjusted	per million
1	12-Apr-20	4,141		0.05 (0.01–0.19)	0.26 (0.03–1.00)	1
2	25-Apr-20	4,460		0.13 (0.06–0.30)	0.61 (0.19–1.45)	3
3	09-May-20	4,500		0.22 (0.12–0.41)	0.94 (0.39–1.90)	8
4	23-May-20	4,500		0.18 (0.09–0.35)	0.74 (0.28–1.58)	15
5	27-Jun-20	4,500		0.47 (0.30–0.72)	2.12 (1.12–3.62)	49
6	25-Jul-20	4,500		0.96 (0.71–1.29)	4.15 (2.55–6.36)	137
7	15-Aug-20	4,500		1.22 (0.93–1.60)	4.82 (3.09–7.12)	233
8	05-Sep-20	4,500		1.38 (1.06–1.79)	5.15 (3.40–7.43)	326
9	06-Feb-21	4,501	9.97 (9.06–10.95)	2.04 (1.64–2.52)	—	965
10	10-Apr-21	4,499	18.16 (16.90–19.49)	—	—	1,922

Table 3 shows a comparison of seropositivity between population subgroups according to vaccination, based on the ELISA results in the last two rounds. As expected, the prevalence of seropositive individuals is systematically higher among those who were already vaccinated. These differences are more marked in the round 10, with a larger proportion of the population already vaccinated and with more time for antibodies to develop. Among individuals aged 80+ years we found 34.0% (95%CI 26.3–42.7) of seropositivity among those vaccinated, compared to 12.5% (95%CI 1.7–53.8) among the unvaccinated. We found similarly high seropositivity among age groups 20–39 and 40–59 which concentrate health professionals that were among the first groups to receive the vaccine. Overall, in round 10, 22.5% (95% CI 20.1–25.2) of the vaccinated individuals were seropositive compared to 16.6% (95%CI 15.2–18.1) of the unvaccinated. For round 9 these proportions were 21.9% (95%CI 14.8–31.2) and 9.7% (95%CI 8.8–10.7) respectively.

**Table 3:** Seroprevalence based on the ELISA test, by sentinel city and by population subgroups. Source: EPICOVID19-RS study, Brazil, 2020–21.

	Round 9			Round 10		
	
	% vaccinated^a^	Vaccinated^a^	Unvaccinated	% vaccinated^a^	Vaccinated^a^	Unvaccinated
City		p = 0.2729	p = 0.4824		p = 0.7264	p = 0.2908
Canoas	1.4	28.6 (7.2–67.4)	12.4 (9.4–16.1)	22.2	20.4 (12.9–30.6)	18.4 (14.1–23.7)
Caxias do Sul	0.8	25.0 (3.3–76.3)	9.3 (7.0–12.3)	22.4	24.3 (17.4–32.9)	15.2 (11.6–19.6)
Ijuí	1.8	55.6 (25.1–82.4)	9.4 (7.0–12.4)	27.8	23.2 (17.0–30.8)	16.2 (12.1–21.2)
Passo Fundo	2.4	25.0 (8.8–53.4)	10.9 (8.2–14.2)	25.9	22.0 (15.5–30.4)	16.2 (11.5–22.2)
Pelotas	2.6	15.4 (4.0–44.3)	8.7 (6.4–11.6)	29.6	23.0 (16.5–31.0)	13.8 (10.1–18.5)
Porto Alegre	3.4	5.9 (0.8–32.3)	8.4 (6.4–11.0)	27.2	23.5 (17.1–31.4)	15.4 (11.9–19.8)
Santa Cruz do Sul	2.2	27.3 (9.0–58.6)	7.9 (5.8–10.7)	26.4	28.2 (20.2–38.0)	14.0 (11.0–17.8)
Santa Maria	2.2	22.2 (5.6–58.0)	10.0 (7.6–13.0)	27.6	16.4 (11.1–23.7)	19.2 (15.6–23.3)
Uruguaiana	2.8	14.3 (3.8–41.0)	10.4 (7.3–14.5)	25.6	21.6 (14.1–31.6)	21.0 (16.8–25.9)
Sex		p = 0.2405	p = 0.2440		p = 0.0620	p = 0.1115
Male	1.3	13.0 (4.3–33.6)	9.0 (7.8–10.5)	22.3	19.2 (15.7–23.3)	15.4 (13.5–17.5)
Female	2.7	24.7 (16.3–35.5)	10.1 (8.9–11.5)	28.4	24.2 (21.0–27.7)	17.4 (15.7–19.4)
Age (years)		p = 0.1825	p = 0.2533		p = < 0.001	p = 0.5704
0–10	0	—	9.2 (4.9–16.7)	0	—	21.8 (14.3–31.8)
11–19	0	—	9.8 (6.5–14.4)	0.5	100	17.4 (12.8–23.1)
20–39	4.1	18.2 (9.2–32.7)	11.3 (9.5–13.4)	11.0	31.9 (23.9–41.2)	17.2 (14.9–19.8)
40–59	2.5	32.4 (19.7–48.4)	10.0 (8.5–17.8)	6.0	37.4 (27.6–48.3)	16.4 (14.4–18.4)
60–79	0.9	8.3 (1.2–41.4)	8.4 (7.0–10.1)	66.5	16.8 (14.3–19.7)	14.3 (11.2–18.1)
80+	1.3	0.0	7.7 (4.8–12.3)	94.3	34.0 (26.3–42.7)	12.5 (1.7–53.8)
Skin color		p = 0.0783	p = 0.0262		p = 0.0036	p = 0.1768
White	2.3	17.8 (10.7–28.2)	9.1 (8.1–10.2)	27.7	21.8 (19.1–24.8)	15.6 (14.0–17.2)
Brown (“*pardo*”)	2.3	37.5 (18.9–60.8)	10.3 (8.2–12.9)	20.7	21.4 (15.4–28.9)	18.9 (15.7–22.6)
Black	1.9	16.7 (2.3–63.2)	14.3 (11.3–18.1)	21.4	29.6 (20.4–40.9)	19.4 (15.0–24.6)
Yellow or Asian	0	—	6.9 (1.7–24.1)	32.1	33.3 (11.1–66.7)	10.5 (2.6–33.8)
Indigenous	3.9	100	16.7 (5.6–40.2)	33.3	83.3 (36.9–97.7)	25.0 (8.0–56.1)
Schooling		p = 0.4534	p = 0.0258		p = 0.1321	p = 0.3821
Primary (0–4 years)	0	—	8.7 (5.7–12.9)	59.4	12.8 (7.7–20.6)	18.7 (11.0–29.8)
Primary (5–9 years)	0.6	50.0 (5.9–94.1)	8.0 (5.7–11.1)	38.3	23.1 (17.0–30.7)	13.5 (9.8–18.3)
Secondary	0.3	0.0	11.9 (9.4–14.9)	26.5	21.3 (15.3–28.8)	16.2 (12.8–20.3)
Higher (incomplete)	2.0	34.8 (18.4–55.8)	11.4 (9.7–13.3)	22.2	25.4 (20.7–30.8)	18.6 (16.2–21.3)
Higher (complete)	3.1	22.0 (12.7–35.3)	8.5 (7.2–10.0)	22.8	22.6 (18.1–27.7)	16.7 (14.5–18.9)
Wealth quintiles (IEN)		p = 0.0228	p = 0.9245		p = 0.3003	p = 0.3028
Q1 (poorest)	1.4	15.4 (3.9–45.1)	9.6 (7.8–11.8)	34.4	21.8 (17.6–26.6)	18.6 (15.7–21.9)
Q2	1.8	17.7 (5.8–42.8)	10.1 (8.4–12.1)	22.4	27.6 (21.5–34.7)	16.7 (13.9–19.7)
Q3	2.4	50.0 (28.5–71.5)	10.0 (8.1–12.3)	20.8	21.9 (16.5–28.4)	14.5 (11.9–17.6)
Q4	2.1	15.8 (5.2–39.2)	10.0 (8.1–12.3)	25.3	23.4 (18.2–29.5)	17.6 (14.9–20.7)
Q5 (richest)	3.4	11.1 (3.6–29.7)	8.9 (7.0–11.3)	25.6	18.5 (13.5–24.6)	15.7 (13.0–18.9)
Exposure score (deciles)		p = 0.2686	p = 0.0393		p = 0.2759	p = 0.2207
D1 (least exposed)	1.0	0.0	8.1 (5.4–12.0)	45.9	19.8 (14.5–26.3)	15.9 (11.3–21.9)
D2	1.3	0.0	10.8 (7.7–15.0)	36.3	23.6 (17.4–31.1)	16.4 (12.6–21.0)
D3	1.0	0.0	8.5 (6.4–11.2)	29.2	25.8 (19.0–33.9)	13.2 (9.8–17.7)
D4	0.6	0.0	8.1 (6.1–10.7)	28.3	20.5 (13.8–29.4)	17.9 (13.8–22.8)
D5	0.7	0.0	9.1 (6.2–13.3)	27.0	20.4 (13.4–29.9)	15.5 (11.6–20.4)
D6	1.9	30.0 (10.0–62.4)	9.0 (6.8–11.7)	23.0	18.9 (12.6–27.2)	16.1 (12.8–20.0)
D7	3.1	12.3 (3.1–38.7)	8.2 (6.0–11.1)	21.1	26.4 (18.8–35.7)	17.4 (13.8–21.7)
D8	3.0	18.8 (6.2–44.8)	9.9 (7.6–12.9)	21.6	22.7 (15.7–31.6)	15.2 (11.9–19.1)
D9	3.6	42.1 (22.8–64.1)	10.7 (8.3–13.7)	17.2	19.8 (12.3–30.3)	15.9 (12.6–19.9)
D10 (most exposed)	4.2	27.8 (12.4–51.1)	14.4 (11.6–17.8)	18.5	39.1 (25.9–54.2)	23.9 (18.3–30.6)

^a^ Vaccinated with one or two doses of any of the SARS-CoV-2 vaccines.

The last round of the study took place 12 weeks after the start of COVID-19 vaccination. The priority groups for vaccination included front line health workers, indigenous people, and older people, in decreasing order of age. Nearly 95% of older people aged 80 years or more were already vaccinated by early April with at least one dose, as did two thirds of those aged 60–79 years (Table 3). Indigenous people were also included in the priority groups, although only 33.3% (95%CI 16.1–56.5) of the 18 individuals in the sample reported having been vaccinated.

Across subgroups, the only notable differences are related to ethnicity based on skin color. In round 9 we see that unvaccinated indigenous and black individuals present much higher seropositivity compared to whites. In round 10 we see a similar pattern (although the differences are no longer significant). Among the vaccinated indigenous individuals in round 10 we found the highest seropositivity in all groups, 83.3% (95%CI 36.9–97.7), albeit with a very wide confidence interval given there are just six vaccinated individuals here).

**Figure 2:**
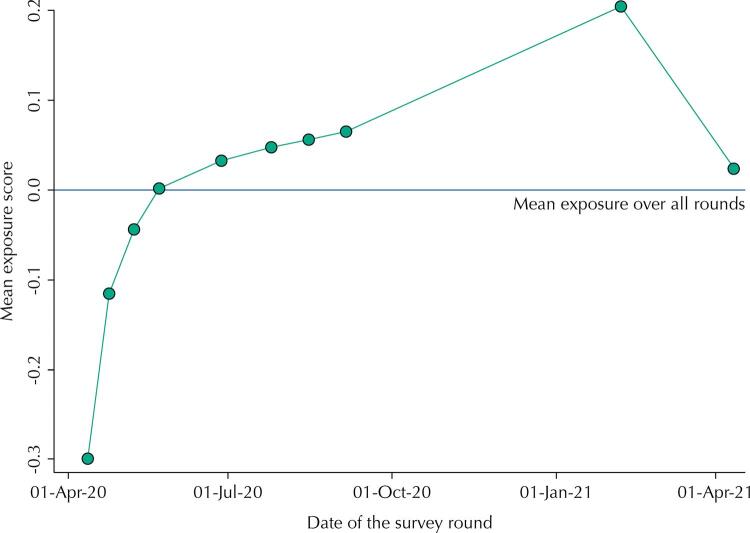
Mean exposure score for the ten rounds of the EPICOVID19-RS study. Source: EPICOVID19-RS study, Brazil, 2020–21.

**Figure 3:**
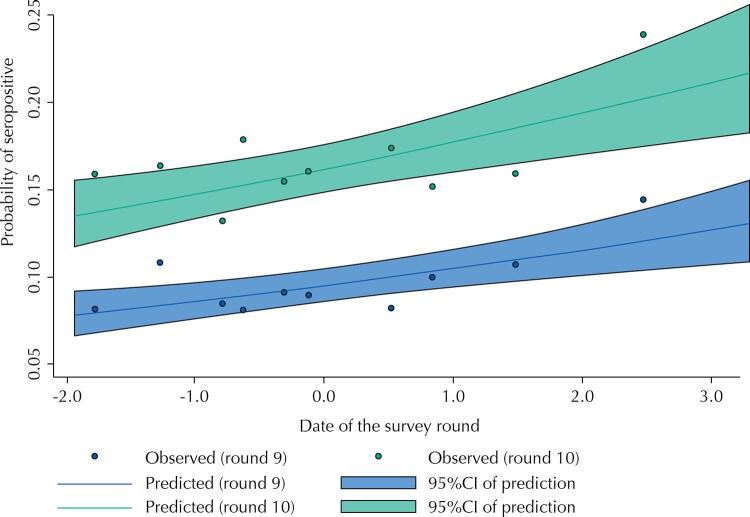
Probability of being seropositive according to the exposure score, estimated through a logistic regression model (7.8% to 13.0%, from the lowest to the highest exposure for round 9 and from 13.5% to 21.7% for round 10). The observed percentages of seropositives for each exposure decile are presented as dots. Source: EPICOVID19-RS study, Brazil, 2020–21.

The exposure score that was generated by PCA had a mean of zero (by construction) that represents the average value of exposure along the study period, with a standard deviation of 1.25. The first component used to derive the score explained 52% of the total variability of the three social distancing indicators. Figure 2 shows a rapid increase in exposure from the first to the fourth round of the study, covering the first five months of the pandemic. After that we see a slower but steady increase in the exposure score until January 2021, when it decreases to a value close to zero, the average level of exposure observed in the study. This final decrease in exposure followed the huge spike of cases and deaths in February and March. We also found a clear association at the individual level between the exposure score and seropositivity during the last two rounds (Figure 3). Using the continuous exposure score as the predictor, we used a logistic regression model to predict the seroprevalence for the average scores in each decile for the last two rounds. Based on the model, the probability of being seropositive increased from 7.8% to 13.0%, from the lowest to the highest exposure level for round 9 and from 13.5% to 21.7% for round 10. The Hosmer-Lemeshow goodness of fit test yielded a p-value of 0.7518 indicating the model is adequate, without suggestion of non-linearity or interaction in the logit-linear model.

## DISCUSSION

Our study, to our knowledge, is the longest series of surveys carried out on the same population, covering 10 rounds over 13 months of the COVID-19 pandemic. Earlier results for phases 1–8 were published elsewhere^10^. At the time of the early rounds of the study, the only rapid test available in large amounts in the country was the Wondfo lateral-flow test, which had been imported by a Brazilian mining conglomerate and donated to the Ministry of Health. A set of early validation studies, including our own, suggested that the test had sensitivity above 80% in subjects who had been recently diagnosed using RT-PCR – given that all cases in the country were recent as of April 2020. Over time, the literature started showing that the sensitivity of several different antibody tests declined with time since the infection, and we decided to carry out a second validation study that confirmed the decline. This second study provided parameters for adjusting results from the original antibody test^14^ and allowing a comparison with a recent ELISA test developed in Brazil that showed consistently high sensitivity over time since the diagnosis^11^.

As observed in previous studies, some population groups are much more vulnerable to COVID-19, especially indigenous and black participants. This reinforces the very different vulnerability of ethnic groups, which is most likely related to macro determinants such as living conditions, family structures, and social norms. Surprisingly, we found no important differences across wealth quintiles, despite indigenous and blacks being, on average, poorer than other ethnic groups. In previous analyses, we found decreasing levels of seropositivity with wealth^5,19^, but at a much lower level, less than 3% at the time, between May and June 2020. The higher seropositivity allied with the changing pattern of exposure may have masked this difference in a crude analysis. On the other hand, the higher seropositivity observed among indigenous and black people has been consistent across the studies^5,19^.

We found a clear increase in seropositivity in relation to our exposure score, highlighting the importance and effectiveness of social distancing, which has been questioned by some activist groups. The 10% of the population most exposed presented a 40% higher seropositivity suggesting that if we could, at a minimum, reduce the exposure to the level of the 10% least exposed, thousands of cases and deaths could be avoided.

The limitations of our analyses include the low participation of children, including adolescents, with only 6.5% individuals 0–19 years in the round 10 sample, probably due to children’s reluctance to undergo a finger prick. Also, the results of our sampling design are not representative of the state population. However, using the main cities in each subregion of the state gives us a precise idea of seroprevalence levels, especially since we did not observe important differences across sites. The decaying sensitivity of the rapid test is another important limitation that we dealt with by the adjustment process that seems to have produced better and credible results, when analyzed over time.

Our results should be used to inform policy. We show that antibody prevalence increased from under 1% in April 2020 to 18% by April 2021. Rio Grande do Sul state was relatively preserved in the early stages of the pandemic in comparison with most other states in the country, and the increase in seroprevalence mirrored the rise in mortality rates (Figure 1). Federal government policy in Brazil has not been evidence-based. Whereas virtually all scientists recommend social distancing, selective lockouts and use of face masks in public, President Bolsonaro has repeatedly stated that such measures are not needed, and that the natural solution to the pandemic is to allow infections to spread until natural herd immunity is reached. Although initial estimates of R zero had set a level of 60–70% prevalence as needed for herd immunity, the appearance of new, more infective variants such as P.1 and more recently an India-originated one, the recognition that immunity is short lived and vaccines have efficacies that may be relatively low, has led to upward corrections in the level of prevalence required for controlling transmission, to 80% or higher^20^. Whatever the required level, our results show that the proportion of the population already infected by SARS-Cov-2 in the state is still very far from any perspective of herd immunity. With about 16% of the unvaccinated population infected, the number of deaths per million reached about 2,000, totaling over 20 thousand deaths in a state with 11.3 million inhabitants. In a simple calculation – ignoring the protection afforded by vaccination – to reach 80% antibody prevalence one would need to multiply the number of deaths by five, with a cumulative total of 100 thousand deaths. Even if this simple calculation based on seroprevalence fails to consider other sorts of immune response – including cellular immunity – the numbers are staggering. The cost of a permissive “natural herd immunity policy” as favored by the federal government in terms of lives lost is clearly unacceptable.
